# Crystal structure of endo-β-1,6-galactanase from *Streptomyces avermitilis*

**DOI:** 10.1107/S2059798326006133

**Published:** 2026-07-21

**Authors:** Zui Fujimoto, Naomi Kishine, Toshihisa Kotake, Satoshi Kaneko

**Affiliations:** a Research Center for Advanced Analysis National Agriculture and Food Research Organization 2-1-2 Kannondai Tsukuba305-8518 Japan; b Department of Biochemistry and Molecular Biology, Graduate School of Science and Engineering Saitama University 255 Shimo-okubo, Sakura-ku Saitama338-8570 Japan; c Department of Subtropical Biochemistry and Biotechnology, Faculty of Agriculture University of the Ryukyus 1 Senbaru Nishihara903-0213 Japan; Station Biologique de Roscoff, France

**Keywords:** endo-β-1,6-galactanases, enzyme–substrate complex, glycoside hydrolase family 30, *Streptomyces avermitilis*

## Abstract

Endo-β-1,6-galactanases hydrolyze β-1,6-linked galactosyl linkages in galactans, yielding β-1,6-linked galacto-oligosaccharides, predominantly galactobiose. Here, we report to our knowledge the first crystal structure of an endo-β-1,6-galactanase, together with its β-1,6-galactobiose-bound complex, revealing the structural basis for substrate recognition by this enzyme. Endo-β-1,6-galactanase from *Streptomyces avermitilis* (*Sa*16Gal30A) is a member of glycoside hydrolase family 30 (GH30) subfamily 5. *Sa*16Gal30A consists of two structural domains: a catalytic (β/α)_8_-barrel domain and an appended β-sandwich domain. The β-1,6-galactobiose complex structure revealed two β-1,6-galactobiose molecules bound within the catalytic domain: one at the catalytic site and another at the distinct surface site distal to the catalytic center. This structure represents the first reported structure of a GH30 subfamily 5 enzyme and provides structural insights into the molecular basis of β-1,6-galactan recognition within the catalytic cleft. *Sa*16Gal30A possesses three extended regions, loops 2, 4 and 8, in the catalytic domain compared with enzymes from other GH30 subfamilies, and these loops appear to modulate substrate specificity towards β-1,6-galactan by shaping the architecture of the catalytic cleft. In addition, a secondary β-1,6-galactan-binding site was identified at a distal location, which may function as a distal subsite, thereby facilitating the efficient hydrolysis of long β-1,6-galactan chains.

## Abstract 2

The crystal structure of *S. avermitilis* endo-β-1,6-galactanase belonging to GH30 subfamily 5 reveals the first structural framework for endo-β-1,6-galactanase. The β-1,6-galactobiose-bound complex identifies the catalytic subsites and a distal secondary sugar-binding site, providing insight into β-1,6-galactan recognition.

## 1. Introduction

Arabinogalactan proteins (AGPs) are cell-surface glycoproteins present in tree exudate gums (Aspinall, 1969 ▸; Showalter, 2001 ▸). They consist of a relatively small protein backbone extensively decorated with arabinogalactan polysaccharides, which are predominantly composed of a β-1,3-galactan main chain, with β-1,6-galactan side chains further substituted with l-arabinose, glucuronic acid and other sugars (Showalter & Basu, 2016 ▸). AGPs are implicated in a wide range of plant biological processes, including cell expansion, cell–cell communication, embryogenesis and responses to biotic and abiotic stresses (Ellis *et al.*, 2010 ▸; Ma & Johnson, 2023 ▸).

The carbohydrate moieties of AGPs are thought to be degraded by bacteria and fungi in nature. Owing to their structural complexity and highly branched architecture, AGP degradation requires the concerted action of multiple glycoside hydrolases (GH) with distinct linkage specificities (Hu *et al.*, 2025 ▸; Knoch *et al.*, 2014 ▸). Exo-β-1,3-galactanase (EC 3.2.1.145), which hydrolyzes the β-1,3-galactan main chain, has been isolated from fungi such as *Irpex lacteus*, *Fusarium oxysporum* and *Phanerochaete chrysosporium* (Tsumuraya *et al.*, 1990 ▸; Okawa *et al.*, 2013 ▸; Ichinose *et al.*, 2005 ▸; Matsuyama *et al.*, 2020 ▸), as well as from bacteria including *Clostridium thermocellum*, *Sphingomonas* sp. and *Bifidobacterium longum* (Ichinose, Kuno *et al.*, 2006 ▸; Sakamoto *et al.*, 2011 ▸; Fujita *et al.*, 2014 ▸). Endo-β-1,6-galactanase (EC 3.2.1.164), which specifically hydrolyzes the β-1,6-galactosyl side chains of AGPs, has also been identified in fungi such as *Trichoderma viride* and *F. oxysporum* (Okemoto *et al.*, 2003 ▸; Sakamoto *et al.*, 2007 ▸), as well as in *Arabidopsis* (Gistelinck *et al.*, 2025 ▸). *Streptomyces avermitilis* and *B. longum* utilize gum arabic as a carbon source and are known to produce both exo-β-1,3-galactanase and endo-β-1,6-galactanase, which hydrolyze the main and side chains of AGPs, respectively (Ichinose, Kotake *et al.*, 2006 ▸, 2008 ▸; Ichinose, Yoshida *et al.*, 2008 ▸; Ichinose *et al.*, 2009 ▸, 2013 ▸; Fujita *et al.*, 2019 ▸). In addition, these organisms produce various other glycoside hydrolases that act on auxiliary sugars present in arabinogalactan (Ichinose, Kotake *et al.*, 2006 ▸, 2008 ▸; Ichinose, Yoshida *et al.*, 2008 ▸; Ichinose *et al.*, 2009 ▸, 2013 ▸).

*S. avermitilis* is an actinomycete originally isolated as a producer of the potent anthelmintic agent avermectin (Burg *et al.*, 1979 ▸; Ikeda *et al.*, 2003 ▸). The endo-β-1,6-galactanase from *S. avermitilis* NBRC14893 (*Sa*16Gal30A) catalyzes the hydrolysis of β-1,6-linked galactosyl linkages in oligosaccharides and polysaccharides, producing galactose and β-1,6-linked galacto-oligosaccharides, predominantly β-1,6-galactobiose, from β-1,6-galactan chains (Ichinose, Kotake *et al.*, 2008 ▸). According to the CAZy database (Lombard *et al.*, 2014 ▸), *Sa*16Gal30A belongs to glycoside hydrolase family 30 (GH30). It was previously classified into GH5 prior to the consolidation of glycoside hydrolase families (St John *et al.*, 2010 ▸), as GH5 and GH30 are closely related and belong to the same clan, GH-A. GH30 enzymes exhibit a wide range of catalytic activities, including β-1,4-xylanase (EC 3.2.1.8), β-glucosidase (EC 3.2.1.21), β-xylosidase (EC 3.2.1.37), glucosylceramidase (EC 3.2.1.45) and endo-β-1,6-glucanase (EC 3.2.1.75). Phylogenetic analyses have further subdivided GH30 enzymes into 12 subfamilies, with enzyme members within each subfamily generally exhibiting similar catalytic activities (St John *et al.*, 2010 ▸; Li *et al.*, 2022 ▸; Šuchová *et al.*, 2024 ▸). *Sa*16Gal30A is classified into GH30 subfamily 5 (GH30_5), which primarily comprises endo-β-1,6-galactanases.

To date, three-dimensional structures have been reported for eight GH30 enzymes, with structural data available for subfamilies 1, 3, 4, 7, 8 and 10 (Dvir *et al.*, 2003 ▸; Kim *et al.*, 2009 ▸; St John *et al.*, 2011 ▸, 2014 ▸, 2022 ▸; Urbániková *et al.*, 2011 ▸; Sainz-Polo *et al.*, 2014 ▸; Freire *et al.*, 2016 ▸; Temple *et al.*, 2017 ▸; Nakamichi, Fujii *et al.*, 2020 ▸; Nakamichi, Watanabe *et al.*, 2020 ▸; Nikolaivits *et al.*, 2021 ▸; Nakamichi *et al.*, 2023 ▸; Vacilotto *et al.*, 2024 ▸). These structures reveal that GH30 enzymes share a conserved basic architecture comprising two domains: a catalytic (β/α)_8_-barrel domain and an appended β-sandwich domain. Some enzymes additionally possess carbohydrate-binding modules, resulting in a modular protein architecture. GH30 enzymes are members of clan GH-A, which is characterized by the (β/α)_8_-barrel fold and a retaining double-displacement catalytic mechanism involving two conserved glutamate residues located at the C-terminal ends of the fourth (acid/base) and seventh (nucleophile) β-strands (Henrissat *et al.*, 1995 ▸). The identity of the nucleophilic residue in GH30 enzymes was first proposed based on covalent labeling of human acid β-glucosidase (*Hs*GCase) by a conduritol-B-epoxide ligand at Glu340 (Premkumar *et al.*, 2005 ▸).

Although GH30 enzymes share a common overall structure, they exhibit distinct substrate-recognition mechanisms owing to local structural variations, resulting in diverse substrate specificities. Several endo-β-1,6-galactanases have been biochemically characterized; however, no crystal structures of an endo-β-1,6-galactanase have been reported to date. In this study, we determined the crystal structure of *Sa*16Gal30A, representing the first three-dimensional structure of an enzyme belonging to the GH30_5 subfamily. This structure provides insights into the molecular basis of β-1,6-galactan recognition and highlights structural features underlying substrate discrimination among GH30 enzymes.

## 2. Materials and methods

### 2.1. *Sa*16Gal30A protein preparation

*S. avermitilis* NBRC14893 was obtained from the National Institute of Technology and Evaluation (NITE; Chiba, Japan). Protein expression and purification were performed as described previously (Ichinose, Kotake *et al.*, 2008 ▸). Briefly, *Escherichia coli* Rosetta (DE3) cells (Merck Novagen) harboring the pET-30+ expression vector (Merck Novagen) containing a truncated *sav_5205* gene encoding residues Thr22–Val491 (GenBank accession No. BAC72917) were cultured for 24 h at 20°C after induction with 0.1 m*M* isopropyl β-d-1-thiogalactopyranoside (IPTG). The recombinant enzyme was purified with nickel–nitrilotriacetic acid–agarose (Qiagen GmbH, Hilden, Germany). Protein concentration was determined by measuring the absorbance at 280 nm, assuming that an absorbance of 1.0 (1 cm path length) corresponds to a concentration of 0.35 mg ml^−1^.

Selenomethionine-labeled (SeMet) *Sa*16Gal30A was expressed in the methionine-auxotrophic *E. coli* strain B834(DE3) (Merck Millipore) using LeMaster medium supplemented with selenomethionine (Nacalai Tesque, Kyoto, Japan) and was purified under the same conditions as the native protein.

### 2.2. Sugar ligands

β-1,6-Galactobiose (Gal_2_) was produced from larch arabinogalactan by enzymatic hydrolysis using the GH43 exo-β-1,3-galactanase from *Phanerochaete chrysosporium* and purified by gel-filtration chromatography with TOYOPEARL HW-40S (Tosoh Bioscience, Tokyo, Japan) as described previously (Okemoto *et al.*, 2003 ▸; Ichinose *et al.*, 2008 ▸; Matsuyama *et al.*, 2020 ▸).

### 2.3. Crystallization of *Sa*16Gal30A 

Wild-type *Sa*16Gal30A was concentrated to 4.6 mg ml^−1^ (*A*_280,1 cm_ = 13.0) by ultrafiltration using a YM-10 membrane filter (Merck Millipore). The protein solution was subsequently filtered through a 0.1 µm membrane filter (Merck Millipore). Crystallization was carried out by the sitting-drop vapor-diffusion method at 293 K using a precipitant solution consisting of 1.6 *M* ammonium sulfate, 10%(*v*/*v*) dioxane (FUJIFILM Wako Pure Chemical) and 0.1 *M* 2-morpholino­ethanesulfonic acid (MES; Dojindo, Kumamoto, Japan) buffer pH 6.5. Plate-shaped crystals with maximum dimensions of approximately 0.2 × 0.3 × 0.03 mm appeared within a few days using 50 µl reservoir solution and drops composed of 1 µl protein solution mixed with 1 µl reservoir solution.

SeMet *Sa*16Gal30A was concentrated to 5.3 mg ml^−1^ (*A*_280,1 cm_ = 15.0) and crystallized using the same sitting-drop vapor-diffusion method at 293 K. Crystals were obtained using a precipitant solution consisting of 20%(*v*/*v*) 2-propanol (FUJIFILM Wako Pure Chemical), 17%(*w*/*v*) PEG 4000 (Hampton Research) and 0.1 *M* sodium citrate buffer pH 5.6 (FUJIFILM Wako Pure Chemical). Thin rod-shaped crystals with maximum dimensions of approximately 0.01 × 0.03 × 0.5 mm appeared within a few days.

### 2.4. Data collection and structure determination

Diffraction experiments for the native and Gal_2_-complex crystals were performed on beamline BL-5A of the Photon Factory (PF), High Energy Accelerator Research Organization, Tsukuba, Japan. To prepare the Gal_2_ complex, 0.5 µl precipitant solution containing approximately 5%(*w*/*v*) Gal_2_, corresponding to 146 m*M*, was added to the native crystal drop and incubated for one day. Crystals were cryoprotected by brief soaking for 10 s in precipitant solution supplemented with 30%(*v*/*v*) ethylene glycol, mounted in 0.3 mm nylon cryoloops (Hampton Research) and flash-cooled under a nitrogen stream at 95 K. Diffraction data were collected at a wavelength of 1.0000 Å using a Quantum 315 CCD detector (Area Detector Systems, Poway, California, USA). Data integration and scaling were performed using *DENZO* and *SCALEPACK* as implemented in the *HKL*-2000 program suite (Otwinowski & Minor, 1997 ▸). Data-collection statistics are summarized in Table 1 ▸.

The crystal structure was determined by the multiple-wavelength anomalous dispersion (MAD) method using selenomethionine-labeled crystals. Diffraction data for these selenomethionine-labeled crystals were collected on beamline AR-NW12A of the Photon Factory Advanced Ring (PF-AR; Chavas *et al.*, 2012 ▸) using a Quantum 210 CCD detector (Area Detector Systems) at three wavelengths: 0.97920 Å (peak), 0.97939 Å (edge) and 0.96400 Å (high remote). A total of 36 selenium sites were identified, and initial phases were calculated using *SOLVE*/*RESOLVE* (Terwilliger, 2002 ▸, 2003 ▸). Automatic model building was carried out using *ARP*/*wARP* (Cohen *et al.*, 2004 ▸) within the *CCP*4 program suite (Winn *et al.*, 2011 ▸; Agirre *et al.*, 2023 ▸). Manual model building and refinement were performed using *Coot* (Emsley & Cowtan, 2004 ▸; Casañal *et al.*, 2020 ▸) and *REFMAC*5 (Murshudov *et al.*, 2011 ▸). The native and Gal_2_-complex structures were determined by molecular replacement using *MOLREP* (Vagin & Teplyakov, 2010 ▸), with the selenomethionine-labeled structure as the search model, and further refined using *REFMAC*5 and *Coot*. Refinement statistics are summarized in Table 2 ▸. Model stereochemistry was validated using *RAMPAGE* (Lovell *et al.*, 2003 ▸).

Structural illustrations were generated using *CueMol*2 (https://cuemol.github.io/cuemol2_docs/en/) and secondary-structure elements were assigned using *DSSP* (Joosten *et al.*, 2011 ▸). The atomic coordinates and structure factors for native Sa16Gal30A, SeMet Sa16Gal30A and the Sa16Gal30A–Gal_2_ complex have been deposited in the Protein Data Bank under accession codes 24td, 24tg and 24te, respectively.

## 3. Results

### 3.1. Overall structure

The crystal structure of *Sa*16Gal30A was determined at a resolution of 1.9 Å, and the structure of its complex with β-1,6-galactobiose (Gal_2_) was determined at a resolution of 2.0 Å. Each structural model contains one protein molecule in the crystallographic asymmetric unit. The N-terminal residues Met6–Arg26 and the seven C-terminal residues Leu493–His499 of the recombinant protein could not be modeled due to a lack of electron density. In addition to sugar and water molecules, the models include sulfate ions, MES and glycerol molecules.

**Figure 1:**
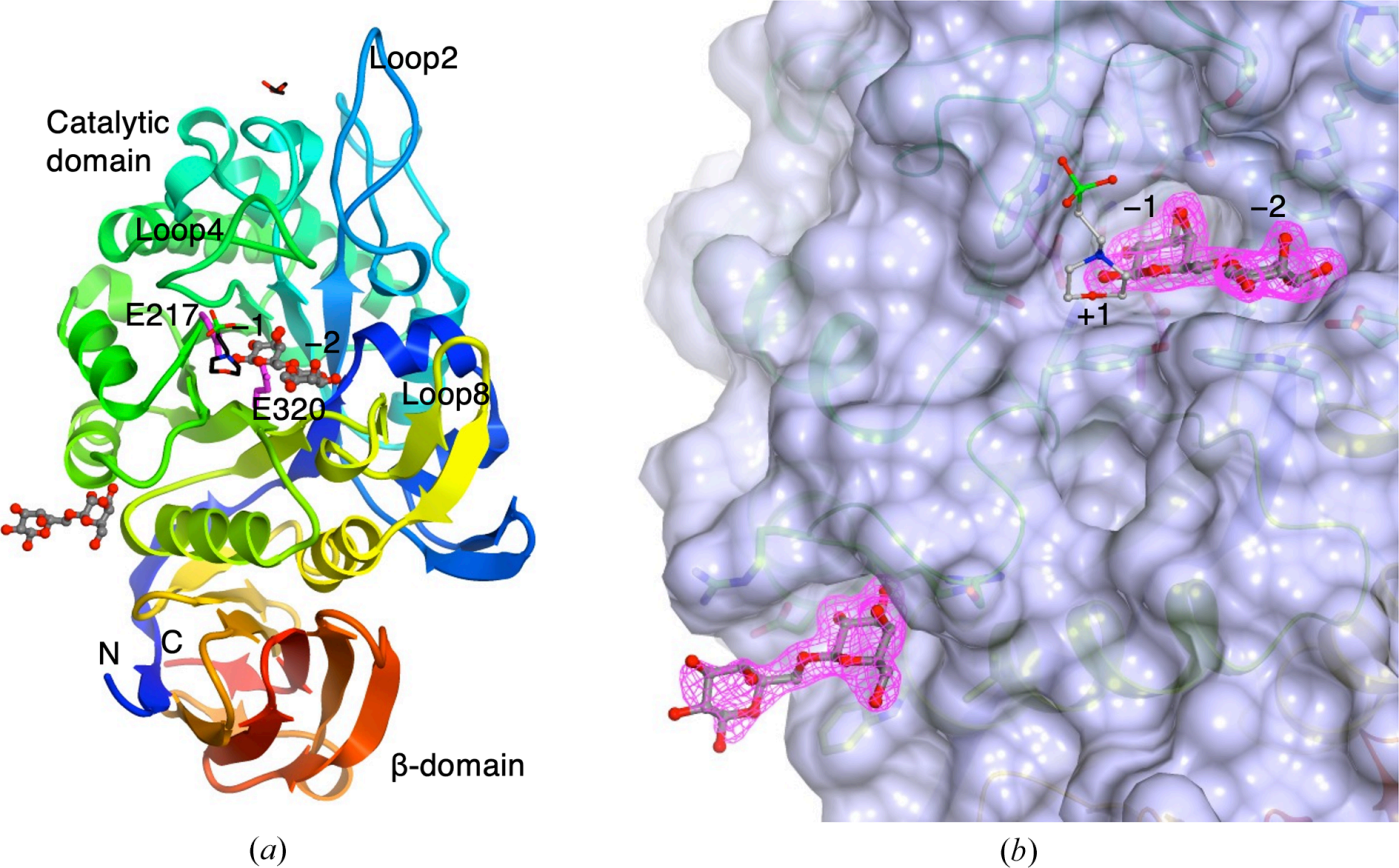
Crystal structure of the *Sa*16Gal30A–Gal_2_ complex. (*a*) Ribbon representation of the *Sa*16Gal30A–Gal_2_ complex, colored from the N-terminus (blue) to the C-terminus (red). Two bound Gal_2_ molecules are shown as ball-and-stick models, and subsites −2 and −1 in the catalytic cleft are indicated. The two catalytic residues are shown as magenta stick models. Bound glycerol and MES molecules are shown as stick models. (*b*) 2*F*_o_ − *F*_c_ electron-density map of the bound Gal_2_ molecules in the catalytic domain, contoured at 1.0σ.

A ribbon representation of the *Sa*16Gal30A–Gal_2_ complex is shown in Fig. 1 ▸(*a*), and the bound Gal_2_ molecules are well defined in the electron-density maps (Fig. 1 ▸*b*). *Sa*16Gal30A consists of two domains: a catalytic domain and a β-sandwich domain. The catalytic domain (Glu43–Ala390) adopts a (β/α)_8_-barrel fold with several extended loops. The β-sandwich domain (Ala29–Trp42, Gly391–Val491) is composed of nine β-strands. The N-terminal β-strand (Thr30–Lys41) forms part of the β-sandwich domain and precedes the catalytic domain. Following completion of the (β/α)_8_-barrel, the remaining β-strands located at the C-terminus assemble to form the rest of the β-sandwich domain. As a result, the mature enzyme both begins and ends within the β-sandwich domain.

**Figure 2:**
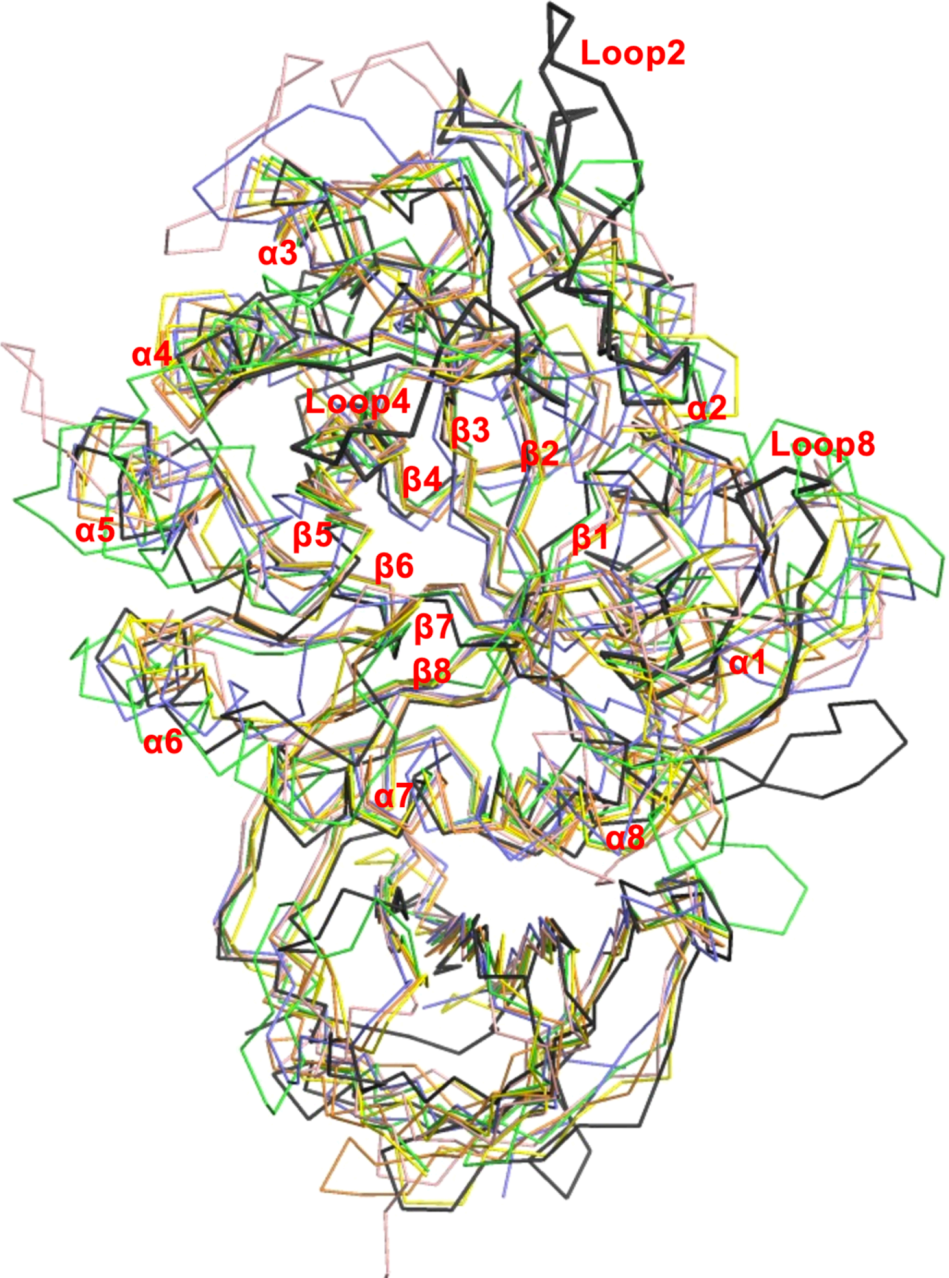
Superposition of the C^α^ traces of the two core domains of representative GH30 enzymes. *Sa*16Gal30A (PDB entry 24td, GH30_5) is shown in black, human acid β-glucosidase (*Hs*GCase; PDB entry 1y7v, GH30_1) in cyan, *Bacteroides thetaiotaomicron* VPI-5482 endo-β-1,6-glucanase BT3312 (PDB entry 5ngl, GH30_3) in blue, an uncharacterized *Bacteroides fragilis* protein BF1510 (PDB entry 3clw, subfamily GH30_4) in green, *Talaromyces cellulolyticus* endo-glucuronoxylanase Xyn30B (PDB entry 6krn, GH30_7) in pink, *Dickeya chrysanthemi* glucuronoxylanase XynA (PDB entry 2y24, GH30_8) in orange and *Acetivibrio clariflavus* xylobiohydrolase (AcXbh30; PDB entry 7n6o, GH30_10) in yellow. The C^α^ traces of the catalytic domains and the attached β-sandwich domains are shown. Signal peptides, tags and additional carbohydrate-binding modules are omitted. Secondary-structure elements forming the (β/α)_8_-barrel are indicated by red letters. The second, fourth and eighth loops of *Sa*16Gal30A are labeled.

The overall structure of *Sa*16Gal30A is conserved among GH30 enzymes (Fig. 2 ▸). The relative positioning of the two domains and the arrangement of secondary-structure elements are maintained, with the exception of slight differences in the organization of β-strands within the β-sandwich domain compared with GH30_1 enzymes, such as human acid β-glucosidase (*Hs*GCase) and *Salmonella* β-glucocerebrosidase (Dvir *et al.*, 2003 ▸; Kim *et al.*, 2009 ▸). Superposition of the C^α^ trace of *Sa*16Gal30A with other structurally characterized GH30 enzymes resulted in root-mean-square deviations (r.m.s.d.s) of 1.9–2.5 Å. Notably, insertions and deletions in loop regions are observed among different subfamilies (Fig. 2 ▸ and Supplementary Fig. S1). A characteristic feature of *Sa*16Gal30A is the presence of three extended loops, corresponding to the second, fourth and eighth loops of the (β/α)_8_-barrel. In this respect, the structure resembles that of an uncharacterized *Bacteroides fragilis* protein belonging to the GH30_4 subfamily (PDB entry 3clw). These extended loops are shown below to contribute to the formation of the catalytic cleft.

In the *Sa*16Gal30A–Gal_2_ structure, two Gal_2_ molecules were observed, one located in the catalytic cleft and the other located in a concave region between the sixth and seventh α-helices of the catalytic domain, distant from the catalytic site (Figs. 1 ▸ and 3 ▸).

Comparison of the apo and Gal_2_-bound structures showed no major conformational changes in the overall fold or in the residues forming the catalytic and secondary Gal_2_-binding sites. The Gal_2_ molecules are absent from the apo structure, but the binding-site residues adopt essentially similar conformations, suggesting that Gal_2_ is accommodated by a preformed binding architecture.

### 3.2. Gal_2_-binding structure in the catalytic site

**Figure 3:**
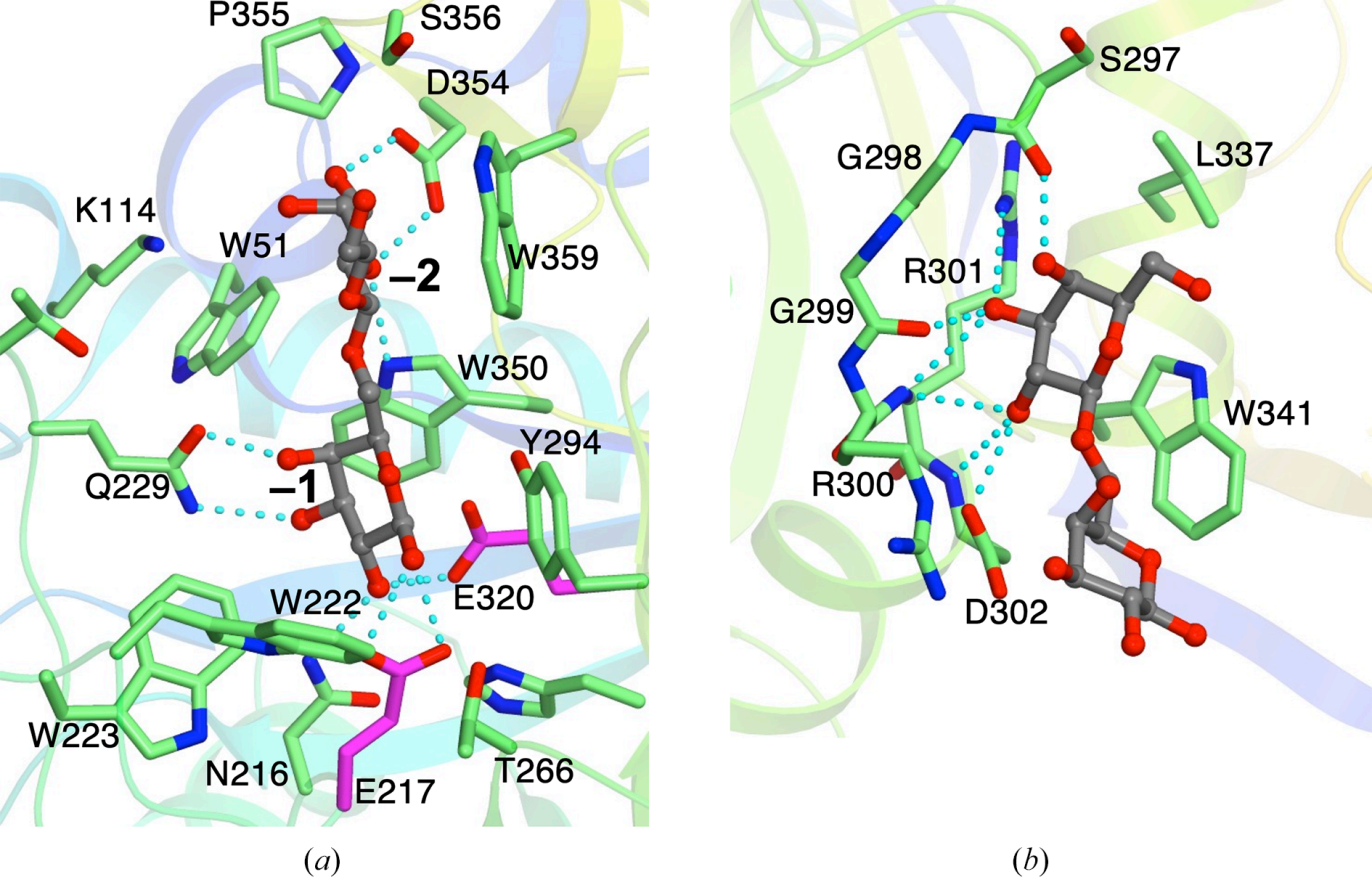
Gal_2_-binding structures in the catalytic domain of *Sa*16Gal30A. **(***a*) Gal_2_ bound at the catalytic site, with subsite labels −2 and −1. Gal_2_ is drawn as a ball-and-stick model, and surrounding protein residues are shown as stick models. The two catalytic residues are shown in magenta. Hydrogen bonds between Gal_2_ and protein atoms are indicated by cyan dashed lines. **(***b*) Gal_2_ bound at the distal binding site.

The catalytic site of *Sa*16Gal30A is located on the C-terminal side of the β-strands within the catalytic domain, where a Gal_2_ molecule is bound in the catalytic cleft. The reducing-end galactose of Gal_2_ is positioned in close proximity to Glu320, which corresponds to Glu340 of *Hs*GCase, a biochemically validated catalytic nucleophile. The distance between the C1 atom of the galactose and the O^ɛ2^ atom of Glu320 is 2.9 Å, consistent with a plausible geometry for nucleophilic attack (Fig. 3 ▸*a*). In addition, the O1 atom of the galactose forms a hydrogen bond with the side chain of Glu217. These observations support the assignment of Glu320 and Glu217 as the catalytic nucleophile and acid/base catalyst, respectively. According to the subsite nomenclature for the glycoside hydrolases (Davies *et al.*, 1997 ▸), the position occupied by the reducing-end galactose corresponds to subsite −1. Glu320 and Glu217 are located at the C-terminal ends of the seventh and fourth β-strands, respectively, consistent with the conserved catalytic residue positioning characteristic of clan GH-A enzymes (Henrissat *et al.*, 1995 ▸).

In addition to the catalytic residues, the galactose at subsite −1 is stabilized by hydrogen bonds to Asn216 and Gln229, as well as by van der Waals interactions with Tyr294 and Trp350. The adjacent galactose at subsite −2, linked via a β-1,6-glycosidic bond, forms hydrogen bonds to Trp350 and Asp354 and is stacked against the aromatic plane of Trp359. The C6 hydroxyl group of this galactose is oriented towards the solvent. Both galactose residues in the bound Gal_2_ molecule adopt the typical ^4^*C*_1_ chair conformation. These inter­actions demonstrate two non-reducing-end subsites within the catalytic cleft of Sa16Gal30A. Additional subsites may exist on the reducing-end side, but further structural or biochemical evidence will be required to define them. On the reducing-end side of the cleft, the pocket is narrowed by the aromatic side chains of Trp222 and Tyr294. Although no galactosyl ligand was observed in this region, a MES molecule was identified in the electron-density map, positioned between these aromatic residues. The morpholine O atom of MES forms a hydrogen bond to the β-anomeric O1 atom of the galactose at subsite −1.

### 3.3. Sugar binding outside the catalytic site

An additional Gal_2_ molecule was observed in a concave region between the sixth and seventh α-helices of the catalytic domain, at a distal site approximately 22 Å from the catalytic center (Fig. 1 ▸). The primary interaction involves the non-reducing-end galactose moiety (Fig. 3 ▸*b*), which is stacked against the indole ring of Trp341 through a face-to-face interaction with the planar sugar ring. This galactose is further stabilized by hydrogen bonds involving the main-chain and side-chain atoms of Arg301 and Asp302, as well as the main-chain atoms of Ser297 and Gly299. The C2 and C3 hydroxyl groups of this galactose are buried within the concave pocket, whereas the C6 hydroxyl group is exposed to the solvent. The reducing-end galactose moiety does not form direct hydrogen bonds to protein atoms, but is positioned near the guanidino group of Arg300 and the carboxylate group of Asp302, which form a salt bridge with each other. Consistent with these observations, the non-reducing-end galactose is tightly bound at this site, as indicated by temperature factors comparable to those of the surrounding protein atoms, whereas the reducing-end galactose appears to be more loosely bound and largely solvent-exposed. This secondary sugar-binding site has not been observed in other structurally characterized GH30 enzymes, and the tryptophan residue responsible for the stacking interaction is not conserved in those enzymes. However, the residues comprising this binding site are conserved among several characterized β-1,6-galactanases belonging to the GH30_5 subfamily (Supplementary Fig. S2), suggesting that this secondary sugar-binding site represents a structural feature of GH30_5 enzymes (Kotake *et al.*, 2004 ▸; Takata *et al.*, 2010 ▸; Li *et al.*, 2022 ▸).

In addition to the two Gal_2_-binding sites described above, one glycerol molecule was found bound at a distal site located between the second and third loops of the catalytic domain (Fig. 1 ▸*a*). The bound glycerol is surrounded by three tryptophan residues (Trp123, Trp165 and Trp185) and three polar residues (His170, Gln183 and Asn186). This aromatic and polar residue-rich environment suggests that this site may represent a potential sugar-binding site. However, no galacto-oligosaccharides were observed at this location under the conditions examined in the present study.

No sugar molecules were observed to bind within the β-sandwich domain of *Sa*16Gal30A. In contrast, in the ligand-bound structure of the GH30 enzyme *Bacillus subtilis* glucuronoxylan xylanohydrolase (*Bs*XynC), a glucuronoxylan is bound within the β-sandwich domain, which appears to function as a carbohydrate-binding domain. In *Bs*XynC, the side chains of Trp376 and His378 stack against the xylan backbone, while Arg353 recognizes the carboxyl group of glucuronic acid. These residues are not conserved in *Sa*16Gal30A, and no sugar-binding site was identified at the corresponding position. Furthermore, no additional candidate sugar-binding sites were detected within the β-sandwich domain of *Sa*16Gal30A.

## 4. Discussion

**Figure 4:**
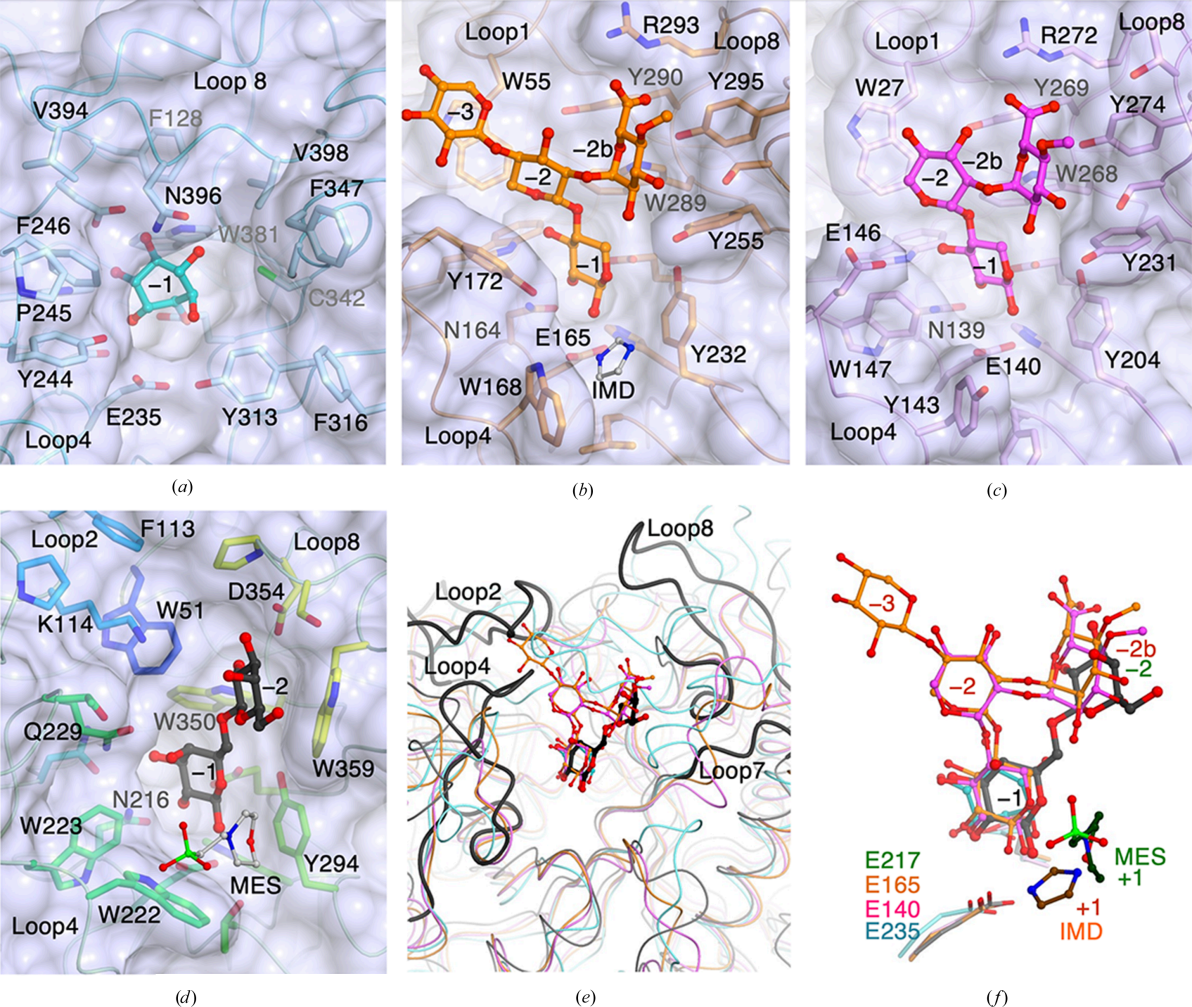
Ligand-binding structures of GH30 enzymes. (*a*) Surface representation of the covalent complex of *Hs*GCase with cyclohexitol (PDB entry 1y7v, GH30_1). The bound conduritol derivative is shown as a cyan ball-and-stick model. (*b*) Surface representation of the MeGX_3_-binding structure of *Dc*XynA (PDB entry 2y24, GH30_8). The bound MeGX_3_ and imidazole molecules are shown as ball-and-stick models in orange and brown, respectively. (*c*) Surface representation of the MeGX_2_-binding structure of *Bs*XynC (PDB entry 3kl5, GH30_8; St John *et al.*, 2011 ▸). The bound MeGX_2_ molecule is shown as a ball-and-stick model in magenta. (*d*) Surface representation of the Gal_2_-binding structure of *Sa*16Gal30A. The bound Gal_2_ and MES molecules are shown as ball-and-stick models with gray and green C atoms, respectively, and protein residues are shown as stick models colored in a rainbow representation. (*e*) Superposition of ligand-binding structures around the catalytic cleft of the *Sa*16Gal30A–Gal_2_ (black), *Hs*GCase–cyclohexitol (cyan), *Dc*XynA–MeGX_3_ (orange) and *Bs*XynC–MeGX_2_ (magenta) complexes. (*f*) Superposition of the bound sugar molecules shown in (*e*). Catalytic residues and the bound MES (dark green) and imidazole (brown) molecules in the *Sa*16Gal30A–Gal_2_ and *Dc*XynA−MeGX_3_ complexes are shown.

The crystal structures of *Sa*16Gal30A (GH30 subfamily 5) provide the first structural information for an endo-β-1,6-galactanase and reveal the molecular basis of β-1,6-galactan recognition at subsites −1 and −2 within the catalytic cleft (Figs. 3 ▸*a* and 4 ▸*a*). Structural analyses of sugar binding are essential for visualizing enzyme subsites and understanding the determinants of substrate specificity. To date, ligand-bound crystal structures have been reported for several GH30 enzymes (Freire *et al.*, 2016 ▸; Lieberman *et al.*, 2007 ▸; Nakamichi *et al.*, 2023 ▸; Nakamichi, Watanabe *et al.*, 2020 ▸; Nikolaivits *et al.*, 2021 ▸; St John *et al.*, 2011 ▸, 2022 ▸; Temple *et al.*, 2017 ▸; Urbániková *et al.*, 2011 ▸); however, no complex structure with a β-1,6-galactan-derived ligand had been obtained prior to this study. In *Hs*GCase, a covalent complex with conduritol-B-epoxide defined the location of subsite −1 and identified the catalytic nucleophile glutamate residue (Fig. 4 ▸*a*; Premkumar *et al.*, 2005 ▸). In the GH30 glucuronoxylanase from *Dickeya chrysanthemi* (synonym *Erwinia chrysanthemi*, *Dc*XynA) complexed with 4-*O*-methyl-aldotetrauronic acid (MeGX_3_), three xylose moieties occupy subsites −1, −2 and −3, while the 4-*O*-methyl-glucuronic acid moiety linked to the xylose at subsite −2 binds to an auxiliary glucuronic acid-specific subsite, termed subsite −2b (Fig. 4 ▸*b*; Urbániková *et al.*, 2011 ▸). Similarly, in *Bs*XynC two xylose moieties of the bound 4-*O*-methyl-aldotriuronic acid (MeGX_2_) occupy subsites −1 and −2, and its 4-*O*-methyl-glucuronic acid moiety binds at subsite −2b, analogous to the *Dc*XynA–MeGX_3_ complex. Another *Bs*XynC complex structure demonstrates that a glucuronic acid molecule alone can occupy subsite −2b (Fig. 4 ▸*c*; St John *et al.*, 2011 ▸). These ligand-binding structures indicate that GH30 glucuronoxylanases recognize their substrates through specific interactions with glucuronic acid or 4-*O*-methyl-glucuronic acid at subsite −2b, requiring substitution of the xylose moiety at subsite −2 for effective substrate recognition.

Structural comparison of the *Sa*16Gal30A–Gal_2_ and *Dc*XynA–MeGX_3_ complexes revealed that the sugar moiety bound at subsite −1 superimposes well between the two structures (Fig. 4 ▸*e*). The catalytic glutamate residues are conserved among GH30 enzymes, and their positions coincide closely in both complexes (Fig. 4 ▸*f*). In each case, the sugar ring at subsite −1 is positioned similarly relative to the catalytic machinery, with distances between the C1 atom of the sugar and the side-chain O atom of the catalytic nucleophile glutamate being less than 3.0 Å, and those between the O1 atom of the sugar and the acid/base glutamate being less than 2.8 Å, indicating equivalent catalytic geometries. In addition to the catalytic residues, other conserved interactions at subsite −1 include an asparagine residue located upstream of the acid/base glutamate (Asn216 in *Sa*16Gal30A and Asn164 in *Dc*XynA), which coordinates the O2 atom of the bound sugar. Hydrophobic interactions involving Tyr294 and Trp350 in *Sa*16Gal30A are also conserved in *Dc*XynA, where they correspond to Tyr232 and Trp289, respectively.

The galactose moiety at subsite −2 in *Sa*16Gal30A does not align with the xylose moiety occupying subsite −2 in the *Dc*XynA–MeGX3 complex. Instead, it aligns more closely with the 4-*O*-methyl-glucuronic acid moiety bound at subsite −2b in *Dc*XynA (Figs. 4 ▸*e* and 4 ▸*f*). This structural correspondence indicates that subsite −2 in *Sa*16Gal30A is positioned analogously to subsite −2b of glucuronoxylanases, despite accommodating a neutral galactose residue rather than an acidic sugar. Key structural differences between galactose and xylose include the orientation of the C4 hydroxyl group and the presence of a C5 hydroxymethyl group. In *Dc*XynA, the O4 atom of xylose is equatorial and participates in formation of a β-1,4-glycosidic linkage with the adjacent xylose residue. In contrast, the O4 atom of galactose in *Sa*16Gal30A is axial and forms a hydrogen bond to the side chain of Gln229, which is located within an inserted region of the fourth loop. This loop is short or absent in *Dc*XynA and other GH30_8 subfamily enzymes. Furthermore, the side chain of Gln229 in *Sa*16Gal30A partially occupies the spatial position corresponding to subsite −2 in *Dc*XynA. This residue therefore appears to discriminate galactose from xylose at subsites −1/–2 by both recognizing the axial O4 hydroxyl group and sterically hindering the formation of a β-1,4-glycosidic linkage. In contrast, galactose possesses a C5 hydroxymethyl group and forms a β-1,6-glycosidic linkage with the adjacent galactose residue. Consequently, the direction of chain extension differs fundamentally between xylan and galactan substrates. As a result, subsite −2 in *Sa*16Gal30A is positioned differently from that in *Dc*XynA and instead resembles subsite −2b, which in glucuronoxylanases specifically accommodates glucuronic or 4-*O*-methyl-glucuronic acid. This rearranged subsite architecture provides a structural basis for the β-1,6-galactan specificity of GH30_5 enzymes.

The 4-*O*-methyl-glucuronic acid moiety at subsite −2b of *Dc*XynA is primarily recognized through polar interactions, including a salt bridge between its carboxyl group and the side chain of an arginine residue. In contrast, the galactose moiety at subsite −2 in *Sa*16Gal30A is recognized through a combination of polar interactions and aromatic stacking inter­actions. Accordingly, the sugar-binding modes at subsite −2 in *Sa*16Gal30A and subsite −2b in *Dc*XynA are markedly different, and the orientations of the bound sugar rings, galactose in *Sa*16Gal30A and 4-*O*-methyl-glucuronic acid in *Dc*XynA, are nearly inverted relative to each other (Fig. 4 ▸*f*). These differences can be attributed to structural variations in the seventh and eighth loops that shape one side wall of the catalytic cleft. The seventh loop of *Sa*16Gal30A is shorter than that of *Dc*XynA, whereas the eighth loop is substantially longer (Fig. 4 ▸*e* and Supplementary Fig. S1). In *Sa*16Gal30A, subsite −2 is formed primarily by residues Trp350, Asp354, Pro355 and Trp359, all of which are located within the elongated eighth loop. In *Dc*XynA, subsite −2b is constructed from residues contributed by both the seventh and eighth loops. Thus, differences in the main-chain architecture of these loops alter the topology of the catalytic cleft and directly influence the sugar-recognition mechanism.

On the opposite side wall of the catalytic cleft, residues Lys114 and Gln229 of *Sa*16Gal30A, originating from the second and fourth loops, occupy the positions corresponding to subsites −2 and −3 in *Dc*XynA, which are involved in recognition of the xylan backbone. *Sa*16Gal30A possesses longer second and fourth loops than enzymes from other GH30 subfamilies, resulting in a narrower catalytic cleft that is well suited for accommodating β-1,6-galactan. In contrast, the shorter loops of *Dc*XynA create a wider catalytic cleft capable of accommodating xylan substrates substituted with glucuronosyl side chains.

A total of four loop regions are primarily involved in formation of the catalytic cleft and contribute to substrate specificity in *Sa*16Gal30A. Among GH30_5 β-1,6-galactanases, these loops are well conserved and show no major insertions or deletions. Outside subfamily 5, sequence alignments indicate that insertions and deletions within these loop regions tend to be conserved within each subfamily, but differ between subfamilies (Supplementary Fig. S1). Because GH30 enzymes share a conserved anomer-retaining catalytic mechanism, including the two catalytic glutamate residues and their surrounding structural framework, variations in the loop regions that form the distal subsites appear to be key determinants of substrate specificity. GH30 enzymes have been classified into multiple subfamilies, each generally exhibiting similar substrate preferences. It is therefore likely that each GH30 subfamily possesses characteristic structural features within these loop regions that define its enzymatic activity and substrate specificity.

Consistent with this view, comparison of the orientations of the catalytic clefts indicates that subfamily 4 enzymes adopt a cleft architecture similar to that of *Sa*16Gal30A, particularly with respect to the relative arrangement of subsites −1 and −2. However, notable differences are observed on the aglycone side of the cleft, suggesting functional divergence despite overall similarity in the glycone binding region. In contrast, enzymes from subfamilies 7, 8 and 10 possess catalytic clefts oriented in a common direction that accommodates extended xylan backbones. Among these, subfamily 10 enzymes lack the glucuronic acid-specific subsite −2b that is characteristic of glucuronoxylanases in subfamily 8, indicating a distinct mode of substrate recognition. By comparison, representative exo-acting enzymes from subfamily 1 and 3 contain closed monosaccharide-recognition pockets and do not possess elongated catalytic clefts suitable for binding linear polysaccharide chains.

The catalytic clefts of *Dc*XynA and *Bs*XynC extend across the catalytic domain and are open at both ends, allowing internal regions of the xylan backbone to dock along the length of the cleft (Figs. 4 ▸*b* and 4 ▸*c*). In contrast, the catalytic cleft of *Hs*GCase forms a closed pocket that accommodates only a single glucose moiety, a characteristic feature commonly observed in exo-acting glycosidases (Fig. 4 ▸*a*). The catalytic cleft of *Sa*16Gal30A more closely resembles a pocket-like structure that accommodates two galactose moieties (Fig. 4 ▸*d*). On the reducing-end side, the cleft is blocked by two aromatic residues: Trp222 and Tyr294. Weak electron density attributable to a MES molecule was observed between these residues, suggesting that this position may correspond to subsite +1, which recognizes the aglycone galactose moiety primarily through interactions with the sugar ring. On the non-reducing-end side, the cleft is also blocked by Pro355 and Ser356 from the eighth loop. The C5 hydroxy­methyl group (O6 atom) of the galactose residue at subsite −2 is oriented towards the solvent, indicating that the adjacent galactose unit on this side likely lies outside the catalytic cleft. Because the C1 and C4 atoms of pyranosyl rings are located on opposite sides of the ring, sugar chains linked by β-1,4-glycosidic bonds tend to adopt relatively linear conformations. Accordingly, xylanases or other glycoside hydrolases that act on β-1,4-linked polysaccharides, such as cellulases and chitinases, typically possess elongated, linear catalytic clefts. In contrast, β-1,6-glycosidic linkages connect the C1 and C5–C6 atoms, which are not positioned opposite each other, and involve rotatable bonds between sugar rings. This geometry results in a more flexible and kinked polysaccharide chain. Owing to this inherent flexibility, *Sa*16Gal30A does not require a linear catalytic cleft to recognize β-1,6-galactan chains. Instead, the enzyme appears to possess two well-defined subsites on the glycone side and likely a more loosely defined subsite on the aglycone side, an arrangement that is well suited for accommodating β-1,6-linked galactan substrates.

In addition to the catalytic cleft, an extra Gal_2_ molecule was observed bound within the catalytic domain at a site approximately 22 Å away from the catalytic center. This secondary binding site is located at the end of the sixth loop and encompasses residues Ser297–Asp302 downstream of Tyr294. Because this secondary site is spatially separated from the catalytic cleft and no continuous ligand density was observed between the two sites, this Gal_2_-binding site may function in a manner similar to a carbohydrate-binding site, contributing to substrate recruitment or to increasing the local concentration of β-1,6-galactan near the catalytic domain. The functional significance of this site should be examined in future studies by mutational analysis of Trp341 and the surrounding residues, combined with activity measurements towards polymeric β-1,6-galactan.

## 5. Conclusions

The crystal structure of *Sa*16Gal30A represents the first reported structure of an endo-β-1,6-galactanase that reveals the structural basis of β-1,6-galactan recognition, and also provides the first structural framework for GH30 subfamily 5. Four conserved loop regions collectively shape the catalytic cleft and play key roles in determining substrate specificity, thereby distinguishing *Sa*16Gal30A from enzymes of other GH30 subfamilies. Structural comparisons with GH30 glucuronoxylanases highlight marked differences in subsite organization and sugar-binding modes, reflecting adaptation to distinct glycosidic linkages. In addition, the presence of a secondary sugar-binding site suggests a potential role in substrate recruitment or enhancement of local substrate concentration. These findings advance our understanding of the enzymatic mechanism of GH30_5 β-1,6-galactanases and provide a structural framework for future functional studies and enzyme engineering.

## Supplementary Material

PDB reference: *Sa*16Gal30A, native (apo), 24td

PDB reference: SeMet, 24tg

PDB reference: complex with Gal_2_, 24te

Supplementary Figures. DOI: 10.1107/S2059798326006133/jc5068sup1.pdf

## Figures and Tables

**Table 1:** Data-collection statistics for *Sa*16Gal30A Values in parentheses are for the highest resolution shell.

	Native	SeMet (peak)	SeMet (edge)	SeMet (high remote)	Gal_2_ complex
Space group	*P*4_3_2_1_1	*P*2_1_	*P*2_1_	*P*2_1_	*P*4_3_2_1_1
*a*, *b*, *c* (Å)	50.1, 50.1, 331.4	43.2, 233.0, 87.9			50.2, 50.2, 330.8
a, β, γ (°)	90, 90, 90	90, 94.9, 90			90, 90, 90
Beamline	PF BL-5A	PF-AR AR-NW12A			BL-5A
Wavelength (Å)	1.0000	0.97920	0.97939	0.96400	1.0000
Resolution range (Å)	100–1.90 (1.97–1.90)	100–2.00 (2.07–2.00)	100–2.00 (2.07–2.00)	100–2.00 (2.07–2.00)	100–2.00 (2.07–2.00)
*R*_merge_	0.144 (0.797)	0.149 (0.392)	0.151 (0.433)	0.161 (0.494)	0.174 (0.907)
*R*_p.i.m._	0.040 (0.217)				0.041 (0.207)
Completeness (%)	100.0 (100.0)	98.4 (98.7)	98.2 (98.4)	97.9 (97.3)	98.6 (100.0)
Multiplicity	13.8 (14.1)	8.2 (8.1)	7.1 (6.9)	7.1 (6.5)	19.6 (19.8)
Average *I*/σ(*I*)	17.0 (2.1)	15.0 (4.8)	13.0 (3.9)	11.6 (3.2)	14.0 (5.6)
Unique reflections	35216 (3426)	114201 (11437)	113947 (11404)	113911 (11322)	29929 (2914)
Observed reflections	486852	938291	813758	807046	587501

**Table 2:** Structure-refinement statistics for *Sa*16Gal30A Values in parentheses are for the highest resolution shell.

	Native (apo)	SeMet	Gal_2_ complex
PDB code	24td	24tg	24te
Resolution range (Å)	49.56–1.90 (1.95–1.90)	43.77–2.00 (2.05–2.00)	49.68–2.00 (2.05–2.00)
*R*_work_	0.152 (0.170)	0.169 (0.180)	0.175 (0.246)
*R*_free_	0.183 (0.185)	0.215 (0.224)	0.234 (0.289)
No. of reflections	33193 (2389)	108230 (8000)	28257 (2060)
No. of water molecules	257	1487	224
Average *B* value (Å^2^)	22.0	14.0	31.5
R.m.s.d. from ideal values			
Bond lengths (Å)	0.004	0.005	0.006
Bond angles (°)	1.24	1.29	1.45
Ramachandran plot			
Favored region (%)	97.8	97.6	97.4
Allowed region (%)	2.2	2.4	2.6
Outlier region (%)	0.0	0.0	0.0

## Data Availability

The atomic coordinates and structure factors for *Sa*16Gal30A have been deposited in the Protein Data Bank under accession codes 24td, 24tg and 24te, and will be released upon publication. All other data supporting the findings of this study are available within the article and its supporting information.
