# Supplementary material for: Crystal structure of endo-β-1,6-galactanase from Streptomyces avermitilis
Source: Acta Crystallogr D Struct Biol. 2026 Jul 21;82(Pt 8):962–71. doi: 10.1107/S2059798326006133 (PMC13431644; doi:10.1107/S2059798326006133)
Supplement: Supplementary file 1 [file d-82-00962-sup1.pdf]

STRUCTURAL
BIOLOGY

Volume 82 (2026)

Supporting information for article:

Crystal structure of endo-β-1,6-galactanase from *Streptomyces avermitilis*

Zui Fujimoto, Naomi Kishine, Toshihisa Kotake and Satoshi Kaneko

```
                                          β1        Loop1        α1
Sa16Gal30A (5)   27 ADATIAVNPSTTYGKWEGWGTSLAWWANVFGA------RDDFADLFFTTKSVTYNGRTLP  80
DcXynA (8)       31 --DTVKIDANVNYQIIQGFGGMSGVGWINDLT-------TEQINTAYGSGV--------G  73
BsXynC (8)        1 MASDVTVNVSAEKQVIRGFGGMNHPAWAGDLT-------AAQRETAFGNGQ--------N  45
AcXbh30A (10)    29 MASTVTVDWDTTYQTIDGFGVSEAFHQSNNIARLGETKQNEIYDLLFSTT---------D  78
TtXyn30A (7)      9 -GTTLTVDLSTTYQRIDGFGTSEAFQRAVQMSRLPEEGQRRALDVLFSTT---------N  58
BT3312 (3)        66 APTTITLNPAEQYQTMDGFGAAITGSTCYNLLLMKPADRHAFLTETFSDK---------D 116
HsGCase (1)      63 TGLLLTLQPEQKFQKVKGFGGAMTDAAALNILALSPPAQNLLLKSYFSEE---------- 112
BF1510 (4)       28 -KKVFIIDKQTVYQEIDNFSASDAWRCAFIGKNWPQEKKEKIADLLFKREFDEK--GNPI  84
```

```
                       β2                  Loop2              ©        α2
Sa16Gal30A (5)   81 GLGLNIARYNLGACSWNSVSGESMVASANIPAFKQIEGYWQDWNNEDP-TSSAWKWTADA 139
DcXynA (8)       74 QIGLSIMRVRIDP-----------------------------------------DSSKWN  92
BsXynC (8)       45 QLGFSILRIHVDE-----------------------------------------NRNNWY  64
AcXbh30A (10)    79 GAGFSIFRSILGDGGTW------------GNADDGPNKTMQPAED------VWDWNESND 120
TtXyn30A (7)     59 GAGLSILRNGIGSSPD--------------MSSDHMVSIAPKSPGSPNNPLIYSWDGSDN 104
BT3312 (3)       117 GFGFSYIRISIGCSDFS----------------LSEYTCCDTKG----IENFALQSEEKD 156
HsGCase (1)     113 GIGYNIIRVPMASCDFS----------------IRTYTYADTPDDF-QLHNFSLPEEDTK 155
BF1510 (4)       85 GMALTNWRVNIGAGSYE-----NREAKEVDNSWNRTECFLSP--------DGKYDFTKQA 131
```

```
                                          β3         Loop3               α3
Sa16Gal30A (5)  140 AQRTMLVKATAR---GATTELFANSPMWWMCLNHNPSGASGGGNNLQ-----SWNYRQHA 191
DcXynA (8)       93 IQLPSARQAVSL---GAKIMATPWSPPAYMKSNNSLIN----GGRLL-----PANYSAYT 140
BsXynC (8)       65 KEVETAKSAVKH---GAIVFASPWNPPSDMVETFNRN-GDTSAKRLK-----YNKYAAYA 115
AcXbh30A (10)   121 DQIPMIRAIQSKY-GVDQILYTVWSPPAWMKNGSVVG------GSLR-----TDKYQAYA 169
TtXyn30A (7)    105 KQLWVSQEAVHTY-GVKTIYADAWSAPGYMKTNGNDANGGTLCGLSGAQCASGDWRQAYA 163
BT3312 (3)      157 YILPILKEILAIN-PSIKVIAAPWTCPKWMKVKSLTDR--TPLDSWTNGQLNPDYYQDYA 213
HsGCase (1)     156 LKIPLIHRALQLAQRPVSLLASPWTSPTWLKTNGAVNG-----KGSLKGQPGDIYHQTWA 210
BF1510 (4)      132 GQQWFMKAARER--GMNNFLFFTNSAPYFMTRSAST-----VSTDQDCINLQNDKFDDFA 184
```

```
                                         β4    © Loop4            α4
Sa16Gal30A (5)  192 SHLAAVALYAKSNWGVNFATVDPFNEPSSSWWTATGTQEGCHMDASVQAAVLPYL-RSEL 250
DcXynA (8)      141 SHLLDFSKYMQT-NGAPLYAISIQNEPDWKPD-----YESCEWSGDEFKSYLKSQ-GSKF 193
BsXynC (8)      116 QHLNDFVTFMKN-NGVNLYAISVQNEPDYAHE-------WTWWTPQEILRFMREN-AGSI 166
AcXbh30A (10)   170 TYLAEHIKNYKSKFGIEITHIGIQNEPNLETS-----YSSCRWSPEELRIFMRDYLVPTF 224
TtXyn30A (7)    164 DYLTKYVEFYQE-SNVTVTHLGFINEPELTTS-----YASMRFSASQAAEFIRIL-YPTI 216
BT3312 (3)      214 TYFVKWIQAFKA-EGIDIYAVTPQNEPLNRGN-----SASLYMEWEEQRDFVKTALGPQM 267
HsGCase (1)     211 RYFVKFLDAYAE-HKLQFWAVTAENEPSAGLLSGYP-FQCLGFTPEHQRDFIARDLGPTL 268
BF1510 (4)      185 RFLVKSAQHFRE-QGFHVNYISPNNEPNGQWHANSF-QEGSFATKADLYRMVEEL-DKAI 241
```

```
                                   β5       Loop5 α5                                     β6
Sa16Gal30A (5)   251  DRRG-LTGTKISASDETSYDL-----------ARTTWGSFG-------SSTKALVNRVNV  291
DcXynA (8)       194  ------GSLKVIVAESLGFNP-------------ALTDPVLKD-----SDASKYVSIIGG  229
BsXynC (8)       167  -------NARVIAPESFQYLK-------------NLSDPILND-----PQALANMDILGT  201
AcXbh30A (10)    225  DKEN--ITAKVVFAENMSFNE-------------QYAINSLND-----PIAVKRVDIVGA  264
TtXyn30A (7)     217  QKSNLTYKPTIACCDAEGWNS-----------QAGMLGALS--------SVNSMFGLVTA  257
BT3312 (3)       268  KAAG--LSTKIYAFDHNYNYD-------NIESQKNYPGKIYED-----AAASQYLAGAAY  313
HsGCase (1)      269  ANST-HHNVRLLMLDDQRLLL------------PHWAKVVLTD-----PEAAKYVHGIAV  310
BF1510 (4)       242  SEAQ--IDTKILIPEVGDMKYLFEIDSIAKTPDDIIHSMFYKDGQYSVLKFKNLFNCVAA  299

                            Loop6         α6           β7   ©          Loop7            α7
Sa16Gal30A (5)   292  HGYQGSGGR-------RDLLYTDVVTTAGKALWNSETGDSDG---------------TGL  329
DcXynA (8)       230  HLYGTTP----------KPYPLAQN--AGKQLWMTEHYVDSK--QSANN------WTSAI  269
BsXynC (8)       202  HLYGTQVSQ--------FPYPLFKQKGAGKDLWMTEVYYPNSDTNSADR------WPEAL  247
AcXbh30A (10)    265  HNYGSSY------------IPFTTTKSKGKGIWMTEVSDMNGNDTT---------INDGL  303
TtXyn30A (7)     258  HAYTSQP----------------GFSMNTPHPVWMTEAADLQGAWTSAWYSYG--GAGEGW  300
BT3312 (3)       314  HNYGGNR----------EELLNIHQAYPEKELLFTETSIGTWNSGRD-------LSKRLM  356
HsGCase (1)      311  HWYLDFLAP------AKATLGETHRLFPNTMLFASEACVGSKFWEQSVRLG---SWDRGM  361
BF1510 (4)       300  HDYWSAYPATLLVDIRNRIHKELSANGHNTKFWASEYCILEKNEEITMPASPERSINLGL  359

                                         β8                    Loop8
Sa16Gal30A (5)   330  TLASNLCLDFRWLHPTAWVYWQVMDPS-----------SGWAMIAYDAS---------TL  369
DcXynA (8)       270  EVGTELNASMVS-NYSAYVWWYIRRS--------------YGLLTE--------------  300
BsXynC (8)       248  DVSQHIHNAMVEGDFQAYVWWYIRRS--------------YGPMKE--------------  279
AcXbh30A (10)    304  RWAKEIHDFMTITEGNAWFYWWGACFK---------TYNGEGLIQMDLN----------S  344
TtXyn30A (7)     301  TWANNVYNAIVNGNASAYLYWIGAQTG----------NTNSHMVHIDAN----------A  340
BT3312 (3)       357  EDMEEVALGTINNWCKGVIVWNLMLDNDRGPNREGGCQTCYGAVDINNS---------DY  407
HsGCase (1)      362  QYSHSIITNLLY-HVVGWTDWNLALNP--EGGPNWVRNFVDSPIIVDITK----------  408
BF1510 (4)       360  YVARIIHNDLTLANASAWQWWTAVSLG------------EDVPIQLLPLEGSNGLSLQYD  407

                         α8
Sa16Gal30A (5)   370  QPGAVQTKYYVMAQFSRHIRAGMTIVDTGVGY------------AAAAYDATARRLVIVA  417
DcXynA (8)       301  -DGKVSKRGYVMSQYARFVRPGALRIQATENPQ-------SNVHLTAYKNT-DGKMVIVA  351
BsXynC (8)       280  -DGTISKRGYNMAHFSKFVRPGYVRIDATKNPN-------ANVYVSAYKGD--NKVVIVA  329
AcXbh30A (10)    345  KTYKVAKRLYTIGQFSRFIRPGWQRIEATKNPV-------SNVYVTAYKDPKTGKFAIVA  397
TtXyn30A (7)     341  GTVEPSKRLWALGQWSRFVRPGARRVAVSGASG--------SLRTAAFRNE-DGSVAVVV  391
BT3312 (3)       408  KTIIRNSHYYIIAHLSSVVKPGAVRIATTGYTD-------NGITCSAFENT-DGTYAFVL  459
HsGCase (1)      409  DTFYKQPMFYHLGHFSKFIPEGSQRVGLVASQK-------NDLDAVALMHP-DGSAVVVV  460
BF1510 (4)       408  GEISTTKMLWTTANYSFFVRPGMKRIAIKPTYKISDLEAATSLMISSYTDG--KEVVTVA  464
```

```
                       .         .         .         .         .
Sa16Gal30A (5)   418 VNTSTSAQTLTFDLSRFSTVTGGTGGLVRRWNTVTGGGGDLYAAHSDTYLSG--------  469
DcXynA (8)       352 VNTNDSDQMLSLNISNANV------TKFEKYSTSA---SLNVEYGGSSQVDS--------  394
BsXynC (8)       330 INKSNTGVNQNFVLQNGSA------SNVSRWITSS---SSNLQPGTNLTVSG--------  372
AcXbh30A (10)    398 INNGWSKQSITYTLKGFSP------ASVTPYTTSS---TQNLEKG-SDITVN--------  440
TtXyn30A (7)     392 INSG-GDAAVNVRLASSSS-ADQQPASAKAWATDN---SRAIEEIQASFAD---------  437
BT3312 (3)       460 INNNEKSKKITVSDG~ 474  37 GDVAIYTTTS-SLTRDLTRDAVNFSPKDNL~ 065
HsGCase (1)      461 LNRSSKDVPLTIKDPA~476  35 GTFSRYESTR--SGRRMELSMGPIQANHTG~ 062
BF1510 (4)       465 INYSKENQVISLNCDHA--------QKGKVYLTTI---DKNLRYMGEQPL----------  504

                       .         .         .
Sa16Gal30A (5)   470 -KSLSVPFAAGAVQTLEVDGVTVK                                      492
DcXynA (8)       395 SGKATVWLNPLSVTTFVSK                                           413
BsXynC (8)       373 NHFWAHLPAQSVTTFVVNR                                           391
AcXbh30A (10)    441 NSSFSFELAPNSITTFVGDTESAS                                      461
TtXyn30A (7)     438 -GVATVNVPSRSMTTVVLYPAAD                                       459
BT3312 (3)       475 QRHFAYDVPGKSVTSYRWAKSK                                        494
HsGCase (1)      477 VGFLETISPGYSIHTYLWHRQ                                         497
BF1510 (4)       505 ---KKLQLPARSVATIVV                                            519
```

Figure S1. Structure-based amino acid sequence alignment of the two core domains of GH30 enzymes. Catalytic residues are indicated by © symbols, and residues of *Sa*16Gal30A that interact with the bound Gal_2 molecule are shown in bold. The secondary structure elements composing the $(\beta/\alpha)_8$-barrel are indicated by magenta for α-helices and green for β-strands and labeled. Amino acid numbers of *Sa*16Gal30A are shown as dots at every ten residues. Abbreviations, GH30 subfamilies and PDB entries: *Dc*XynA (GH30_8, 2Y24), *Dickeya chrysanthemi* glucuronoxylanase; *Bs*XynC (GH30_8, 3KL0), *Bacillus subtilis* glucuronoxylan xylanohydrolase; *Ac*Xbh30A (GH30_10, 7N6O), Acetivibrio clariflavus xylobiohydrolase; *Tt*Xyn30A (GH30_7, 7NCX), *Thermothelomyces thermophilus* glucuronoxylan endo-β-1,4-xylosidase; BT3312 (GH30_3, 5NGL), *Bacteroides thetaiotaomicron* VPI-5482 endo-β-1,6-glucanase; *Hs*GCase (GH30_1, 1Y7W), human acid-β-glucosidase; BF1510 (GH30_4, 3CLW), uncharacterized *Bacteroides fragilis* protein.

```
Sa16Gal30A    1  MIRRRTLLAAAGGTFLGSALATGTARADATIAVNPSTTYGKWEGWGTSLAWWANVFGARDDF   62
Nc16Gal       1  ---MVPSLQTVGHILVTALAIIPFAFADLTTTIDPSSNWGTWEGWGTSLAWWAATFGTRDDL   59
Pc16Gal       1  MIMKQIMNHEWLLGLALTAATIPTVAADLTTTISSTTNWGTWEGFGVSLAWWAKAFGTRTDL   62
Tv16Gal       1  -------MRSIVLPSLALALFSQRARADTTLTIDPTSNWGTWEGWGVSLAWWAKAFGNRDDL   55

Sa16Gal30A   63  ADLFFTTKSVTYNGRTLPGLGLNIARYNLGACSWNSVSGESMVASANIPAFKQIEGYWQDWN  124
Nc16Gal      60  ADIFFTTQTTNLNGVSLPGLGFNIARYNAGASSWNSINGTSMVVSPKMIPSRQIEGFWIDWA  121
Pc16Gal      63  ADIFFSTKSTTYNGQSVPGIGLTIARYNAGACSWNTYNGSTMVVSPDMIASRQMDSYWVDWA  124
Tv16Gal      56  ASVFFSRNNQAVNGQTLPGLGFNIVRYNAGACSNNSYDGSTMVVSPNIKPSRQMDGFWLDWA  117

Sa16Gal30A  125  NEDPTSSAWKWTADAAQRTMLVKATARGA-TTELFANSPMWWMCLNHNPSGAS-GGGNNLQS  184
Nc16Gal     122  STSPSSASWNWSADSAQRAMLSKARSRGANILELFSNSPMWWMCNNHNPSGSDDGSSDNLQS  183
Pc16Gal     125  STSPSSSSWSWSVDANQRNMLSLAKARGATTFELFSNSPPWWMCLNHNPSGSDNGTSNNLQS  186
Tv16Gal     118  SSDPSSSSWNWNVDANQRAMLQKAKANGANIFELFSNSPMWWMCNNHNPSGS--GSSDNLQS  177

                                            ©
Sa16Gal30A  185  WNYRQHASHLAAVALYAKSNWGVNFATVDPFNEPSSSWWTATG-TQEGCHMDASVQAAVLPY  245
Nc16Gal     184  WNYQSHAVYMATVAKYFADHWSTTFTSVDPFNEPSANWWNGKTGTQEGCHFDVSTQATVLNY  245
Pc16Gal     187  WNYDQHAIYLATIAKYAAANWGISFQSVEAFNEPSSAWWNGQTGTQEGCHFDASIQATVIGY  248
Tv16Gal     178  WNYQNHAVYLADIAQHAQQSWRIQFQSVEAFNEPSSSWWTAEG-TQEGCHFDVSTMATVIGY  238

Sa16Gal30A  246  LRSELDRRGLTGTKISASDETSYDLARTTWGSFGSSTKALVNRVNVHGYQGSGGRRDLLYTD  307
Nc16Gal     246  LRAELDSRGLSSTVISASDESYYDQAAKTLQNLGSTALGKIARVNVHGYQYGSGRRDSVRS-  306
Pc16Gal     249  LRTELNNRGLGSTLIAASDETSYDLAITTWNSLGSTATGNVNRINVHGYQYTGGRRDTLYS-  309
Tv16Gal     239  LNTELSSRGLS-SFVASSDENTYDLAISTWQGFNSSTRNIVKRINVHGYQDGGGRRDTLYS-  298

                         ©
Sa16Gal30A  308  VVTTAGKALWNSETGDSDGTGLTLASNLCLDFRWLHPTAWVYWQVMDPSSGWAMIAYDASTL  369
Nc16Gal     307  LATQYSKKLWQSEYGEGDATGRDLASNLLLDLRWLQPTAWVYWQALD-GGGWGLVDADNDKK  367
Pc16Gal     310  LASGAGKRLWNSEYGESDATGQRLVTNLLLDFRWLHPTAWVYWQAID-GGGWGLIDGSNDDL  370
Tv16Gal     299  LASQAGKRLWNSEYGDSDASGKSMYQNLLLDFTWLHPTAWVYWQAID-GAGWGLIVGDNDNL  359

Sa16Gal30A  370  QPGAVQTKYYVMAQFSRHIRAGMTIVDTGVGYAAAAYDATARRLVIVAVNTSTSAQTLTFDL  431
Nc16Gal     368  TIGQPTQKYFVLAQFARHVKPGMRILDGGSDYATAAYDAATKKLVIVAVNWG-SAQYLNFDL  428
Pc16Gal     371  TLGAVSQKYYALAQFARHIRPGMRILDGGSNYTVAAYDSSAKKLVIVAANWG-AAQYLNFDL  431
Tv16Gal     360  TLSSASTKYFVLAQLTRHIRQGMQILTTPDVNTAVAYDAGSQKLVIVTANWG-SAQTITFDL  420

Sa16Gal30A  432  SRFSTVTGGTGGLVRRWNTVTGGG-GDLYAAHSDTYLSGKSLSVPFAAGAVQTLEVDGVTV-  491
Nc16Gal     429  SRFKTA-GVNGAVVKRWATQIGASTGKRYVAASDTYIRGTKFWSYFEKEMVQTFEVEGVVL-  488
Pc16Gal     432  SKFSKP-GASGALVPRWITQTGS--GDQYVSYSDTYMSGTKFWSYFPTGAVQTFEVSNVTL-  489
Tv16Gal     421  TRARTA-GSNGATVPRWSTQTGG--GDQYRSYTDTKINNGKFSASFSSGQVQTFEVSGVVLQ  479
```

Figure S2. Amino acid sequence alignment of GH30_5 enzymes. Catalytic residues of *Sa*16Gal30A are indicated by © symbols, and residues that interact with the bound Gal_2 molecule are shown in bold. Residues of *Sa*16Gal30A that constitute the secondary Gal_2-binding site, and the corresponding residues in other enzymes are enclosed in orange boxes. The secondary structure elements of *Sa*16Gal30A forming the $(\beta/\alpha)_8$-barrel are indicated by magenta (α-helices) and green (β-strands). Abbreviations: *Nc*16Gal, *Neurospora crassa* endo-β-1,6-galactanase (Uniprot Q7S2S4); *Ps*16Gal, *Penicillium subrubescens* arabinogalactan exo-β-1,6-galactobiosidase (Uniprot A0A1Q5UQB8); *Tv*16Gal, *Trichoderma viride* endo-β-1,6-galactanase (Uniprot Q7S2S4).
